# 14-3-3ε acts as a proviral factor in highly pathogenic porcine reproductive and respiratory syndrome virus infection

**DOI:** 10.1186/s13567-019-0636-0

**Published:** 2019-02-28

**Authors:** Shengliang Cao, Fangyuan Cong, Min Tan, Guofei Ding, Jiaqi Liu, Li Li, Yuzhong Zhao, Sidang Liu, Yihong Xiao

**Affiliations:** 1 Department of Fundamental Veterinary Medicine, College of Animal Science and Veterinary Medicine, Shandong Agricultural University, Tai’an, China; 2 Shandong Provincial Key Laboratory of Animal Biotechnology and Disease Control and Prevention, Shandong Agricultural University, Tai’an, China; 3 Shandong Provincial Engineering Technology Research Center of Animal Disease Control and Prevention, Shandong Agricultural University, Tai’an, China

## Abstract

The highly pathogenic porcine reproductive and respiratory syndrome virus (HP-PRRSV) emerged in 2006 in China and caused great economic losses for the swine industry because of the lack of an effective vaccine. 14-3-3 proteins are generating significant interest as potential drug targets by allowing the targeting of specific pathways to elicit therapeutic effects in human diseases. In a previous study, 14-3-3s were identified to interact with non-structural protein 2 (NSP2) of PRRSV. In the present study, the specific subtype 14-3-3ε was confirmed to interact with NSP2 and play a role in the replication of the HP-PRRSV TA-12 strain. Knockdown of *14*-*3*-*3ε* in Marc-145 cells and porcine alveolar macrophages (PAMs) caused a significant decrease in TA-12 replication, while stable overexpression of 14-3-3ε caused a significant increase in the replication of TA-12 and low pathogenic PRRSV (LP-PRRSV) CH-1R. The 14-3-3 inhibitor difopein also decreased TA-12 and CH-1R replication in Marc-145 cells and PAMs. These findings are consistent with 14-3-3ε acting as a proviral factor and suggest that *14*-*3*-*3ε* siRNA and difopein are therapeutic candidates against PRRSV infection.

## Introduction

Porcine reproductive and respiratory syndrome (PRRS), caused by the PRRS virus (PRRSV), is one of the more severe diseases affecting the pig industry worldwide. The manifestation of PRRS includes reproductive failure in pregnant sows and respiratory distress in pigs of all ages [1, 2]. The disease was first reported in North America in 1989, and the causative virus, PRRSV, was isolated in 1991 [3]. In China, the first PRRSV strain was isolated in 1996. Ten years later, the emergence of highly pathogenic PRRSV (HP-PRRSV)—first reported in the southern cities of China [4–6]—caused great economic losses for the swine industry. Therefore, the Chinese government listed HP-PRRSV as a first-class animal infectious disease in 2008. The challenges of prevention have been exaggerated since the emergence of the NADC30-like strain of HP-PRRSV in 2014 [7]. Current commercial PRRSV vaccines do not provide complete protection against infection [8, 9]. Although the NADC30-like strains are not as pathogenic as HP-PRRSV, they are characterized by a high incidence of recombination with other virus strains, which leads to changes in virulence [10–12]. Traditional vaccination apparently cannot meet the requirement of the current PRRSV infection scenario.

PRRSV is an enveloped RNA virus belonging to the order *Nidovirales*, family *Arteriviridae*, and genus *Porartevirus*, along with the lactate dehydrogenase-elevating virus and rat arterivirus 1. Its genome is single-stranded, positive-sense RNA containing 10 open reading frames (ORFs). ORF1a and ORF1ab encode polypeptide proteins 1a and 1ab, respectively, which are later cleaved into 12 non-structural proteins, which take part in the PRRSV life cycle. Non-structural protein 2 (NSP2) is the largest product of this cleavage process. *Nsp2* is the most variable gene in PRRSV and is usually considered as a classification standard for different types or subtypes of the virus. While the *Nsp2* gene of HP-PRRSV contains a 90-base-pair (bp) deletion [4–6], its variant in the PRRSV NADC30-like strain carries a 393-bp deletion [13, 14]. The NSP2 protein contains abundant B cell epitopes and can act as an antagonist of interferon (IFN) production [15]. However, little information is available on its role in PRRSV replication, especially in HP-PRRSV.

14-3-3 proteins are a family of highly conserved acidic proteins which are expressed in all eukaryotic cells. This family of proteins includes seven members (β, ε, η, γ, τ, σ, and ζ), which function as homodimers and heterodimers. These proteins have the ability to bind a multitude of functional regulators of many biological processes by interacting with specific phosphothreonine and phosphoserine motifs, which allows them to regulate the cell cycle, intracellular protein trafficking, apoptosis, DNA-damage response, DNA replication, and transcription [16–18]. The 14-3-3 proteins play a role in virus infection and are considered to be potential biomarkers for HIV-related neurodegeneration [19, 20]. They also affect virus infection by multiple pathways. The 14-3-3 proteins can enhance porcine circovirus type 2 infection in PK-15 cells in the presence of IFN-γ [21] or promote autophagy by interacting with microRNA-30a-5p [22]. They control innate antiviral immunity by regulating the retinoic acid-inducible gene I (*RIG*-*I*) translocon, thereby blocking antiviral signaling [23–25].

Given the complexity and multifunctional nature of the NSP2 protein, we have previously conducted a label-free quantitative proteomics study to identify cellular proteins that potentially interact with NSP2. We found that the 14-3-3 protein family strongly interacts with NSP2 and plays a role in the formation of cellular aggressomes [26]. In the present study, we evaluated the effect of 14-3-3 proteins on HP-PRRSV replication and found that targeting this protein family might be a potential therapeutic strategy against HP-PRRSV infection.

## Materials and methods

### Cells and virus

Marc-145 (PRRSV-permissive cell line derived from African monkey kidney cells) and 293T (human embryo kidney cells) cells were obtained from the China Center for Type Culture Collection (Wuhan, China) and cultured in Dulbecco’s modified Eagle’s medium (Gibco, Langley, OK, USA) supplemented with 10% fetal bovine serum (FBS) (Biological Industries, Beit HaEmek, Israel) at 37 °C in 5% CO_2_ in a humidified incubator. This study used the HP-PRRSV strain TA-12, which was previously isolated by our team and has a 90-bp deletion in the *nsp2* gene (GenBank No. HQ416720). A typical low pathogenic PRRSV (LP-PRRSV) strain, CH-1R, was also used.

Primary porcine alveolar macrophages (PAMs) were isolated from five healthy 8-week-old crossbred weaned pigs (Landrace × Yorkshire) by post-mortem lung lavage. The lungs were washed with phosphate-buffered saline (PBS) 2–4 times until the lavage fluid became clear. The fluid of all five animals was pooled and then centrifuged at 600 × *g* at 4 °C for 10 min to collect the PAMs. The cells were maintained in Roswell Park Memorial Institute 1640 medium with 10% heat-inactivated FBS and penicillin–streptomycin (Solarbio, Beijing, China) at 37 °C in 5% CO_2_ in a humidified incubator. The number of PAMs was adjusted to 2.5 × 10^6^/mL, and the aliquots were frozen in liquid nitrogen. To eliminate differences in PAMs batches from different pigs the triplicates were performed with batches belonging to different pigs in each experiment. The pigs were euthanized using a euthanasia method approved by the Animal Care and Use Committee of Shandong Agricultural University.

### Transfection

Recombinant plasmids GFP-nsp2 and pEGFP-C1 (GFP, green fluorescent protein; EGFP, enhanced GFP) were transfected into monolayer 293T cells using Lipofectamine 2000 (Invitrogen, Carlsbad, CA, USA) in accordance with the manufacturer’s instructions. The cells were collected at 24 h post-transfection for Western blot analyses. Marc-145 cells and PAMs were grown in 6-well cell-culture plates and then transiently transfected with siRNA (Table 1) using a transfection reagent (Lipofectamine^®^ RNAiMAX Reagent; Invitrogen, Carlsbad, CA, USA) in accordance with the manufacturer’s instructions. Knockdown efficiencies were determined by Western blot analysis and quantitative real-time PCR (qPCR). At 24 h post-transfection, the cells were mock infected with DMEM or inoculated with TA-12 at a multiplicity of infection (MOI) of 0.1 and harvested at 0, 12, 24, and 36 hours post-infection (hpi). To analyze the effect of *14*-*3*-*3* knockdown on the cells, the viability of transfected cells was measured by the Cell Counting Kit-8 (CCK-8; Beyotime, Nanjing, China) assay as described below. The infected cells were harvested for assaying 14-3-3 protein expression, viral genome replication, and progeny virus production.

### Confocal fluorescence microscopy

293T and Marc-145 cells were seeded on coverslips and transfected with plasmids pEGFP-C1 and GFP-nsp2, respectively. Marc-145 cells were infected with the HP-PRRSV TA-12 strain at 0.01 MOI. At 24 h post-transfection or post-infection, the cells were fixed with 4% formaldehyde and permeabilized with 0.1% (v/v) Triton X-100 in PBS. The transfected 293T and Marc-145 cells were probed with anti-14-3-3β (Abcam, Cambridge, UK) and anti-14-3-3γ/ε/ζ (Santa Cruz Biotechnology, Dallas, TX, USA) antibodies. The 14-3-3 proteins were visualized using Cy3-goat anti-rabbit immunoglobulin (IgG; Jackson, West Grove, PA, USA).

The HP-PRRSV-infected Marc-145 cells were incubated with anti-NSP2 polyclonal antibodies and visualized using fluorescein isothiocyanate (FITC) goat anti-rabbit IgG. The anti-NSP2 antibodies were prepared by immunizing New Zealand white rabbits with a peptide composed of the N-terminal 180 amino acids of NSP2 (NSP2-180), which had been produced previously [27]. The activity of these antibodies was confirmed by Western blot and immunofluorescent and enzyme-linked immunosorbent assays (data not shown). All probed cells were observed under a fluorescence microscope (Leica, SPE, Buffalo Grove, IL, USA).

### GFP pull-down assay

To determine the specific subtype of 14-3-3 proteins interacting with NSP2, a GFP pull-down assay was performed as described previously [26]. Briefly, 293T cells in 10-cm dishes were transfected with recombinant plasmids GFP-nsp2 and pEGFP-C1 (four dishes per plasmid). The cells were harvested and lysed at 24 h post-transfection. Clarified cell lysates were incubated with GFP-Trap beads (ChromoTek, Munich, Germany) for 2 h at 4 °C, and bound proteins were eluted with 100 µL of 2 × SDS sample buffer.

### Western blot

Cellular proteins from Marc-145, 293T, and PAM cells or samples from the pull-down assay were separated by 10–15% SDS-PAGE and transferred to polyvinylidene difluoride membranes (Millipore Corporation, Bedford, MA, USA) using a Bio-Rad semi-dry transfer apparatus (Bio-Rad Laboratories, Hercules, CA, USA) in accordance with standard procedures. The primary antibodies used for detecting viral and host proteins included the 14-3-3 antibodies mentioned above, a glyceraldehyde 3-phosphate dehydrogenase (GAPDH) antibody, and the monoclonal antibody against PRRSV nucleocapsid protein 6D10 [28]. Horseradish-peroxidase-conjugated anti-mouse or anti-rabbit antibodies were purchased from Jackson (West Grove, PA, USA) for use as secondary antibodies. Protein bands were visualized using the Clarity Western ECL substrate (Bio-Rad).

### Real-time PCR (qPCR)

Total RNA was isolated from Marc-145 cells or PAMs using the GeneJET RNA Purification Kit (Thermo Scientific, Massachusetts, USA) and then reverse transcribed using the ReverTra Ace qPCR RT Kit (Toyobo, Osaka, Japan) in accordance with the manufacturer’s instructions. Cellular genes were quantified by relative-quantification PCR (qPCR), with the abundance of *GAPDH* mRNA being used as an internal reference. Primers targeting the mRNA of PRRSV ORF7 were designed for detecting viral genes by absolute qPCR. All qPCR assays were performed with the ABI Real-Time PCR System (Applied Biosystems, Foster City, CA, USA) using the SYBR Green Realtime PCR Master Mix (Toyobo). The primers used for qPCR assays in this study are listed in Table 1.

**Table 1:** **Primers used for qPCR and PCR amplification**

Types	Name	Sense (5′–3′)	Antisense (5′–3′)
siRNA	Epsilon-29	GCUGAGCGAUACGACGAAATT	UUUCGUCGUAUCGCUCAGCTT
	Epsilon-159	GGAGAAUAAUCAGCAGCAUTT	AUGCUGCUGAUUAUUCUCCTT
	Epsilon-643	GCAGUUGUUACGUGAUAAUTT	AUUAUCACGUAACAACUGCTT
	Beta-21	GCUGGUACAGAAAGCCAAATT	UUUGGCUUUCUGUACCAGCTT
	Beta-138	CUCUGUUGCCUACAAGAAUTT	AUUCUUGUAGGCAACAGAGTT
	N.C	UUCUCCGAACGUGUCACGUTT	ACGUGACACGUUCGGAGAATT
qPCR primers	14-3-3β	TGAGAAGAAGCAGCAGATG	TTCCGATGTCCACAGAGT
	14-3-3ε	CGACGAAATGGTGGAGTC	TGCTGGAATGAGGTGTTT
	PRRSV N gene	AGATCATCGCCCAACAAAAC	GACACAATTGCCGCTCACTA
	GAPDH (swine)	ACTCACTCTTCCACTTTTGATGCT	TGTTGCTGTAGCCAAATTCA
	GAPDH (monkey)	ACCCACTCTTCCACCTTCGACGCT	TGTTGCTGTAGCCAAATTCG

### Establishment of stable cell lines with lentivirus infection

*14*-*3*-*3β/ε* genes were amplified from cDNA derived from Marc-145 cells and subcloned into a modified pWPXLd vector (Addgene, Cambridge, MA, USA) containing a puromycin-resistance gene. The following primers were used for cloning the *14*-*3*-*3β/ε* genes: Lenti-betaF: 5′-CGGGATCCATGACAATGGATAAAAGTGAG-3′, Lenti-betaR: 5′-CCCGAATTCTTAGTTCTCTCCCTCCCCAG-3′ and Lenti-epsilonF: 5′-CGGGATCCATGGATGATCGAGAGGATCTG-3′, Lenti-epsilonR: 5′-CCCGAATTCTCACTGATTTTCGTCTTCCAC-3′. These constructs (or a pWPXLd empty vector) were co-transfected with lentiviral packaging plasmids psPAX2 and pMD2.G (in a 3:2:1 ratio) into 293T cells in 6-well plates (40–50% confluence) using Lipofectamine 2000 in accordance with the manufacturer’s instructions. At 48 h post-transfection, the lentivirus was harvested and filtered using a 0.45 µm filter, mixed with an equivalent volume of complete medium, and infected to monolayers of Marc-145 cells. After incubation for 12 h, the spent medium was replaced with fresh medium. At 48 hpi, 14-3-3β/ε overexpression cells as well as the pWPXLd empty-vector-infected cells were screened using 10 μg/mL puromycin. Marc-145 cells exhibiting stable expression of the β and ε subtypes of 14-3-3 were obtained by subcloning in 96-well plates and named Marc-145^14-3-3β^, and Marc-145^14-3-3ε^, respectively; cells containing the pWPXLd empty vector were termed Marc-145^wpxld^.

### Cell viability assay

The cytotoxicity of difopein was evaluated by the CCK-8 assay in accordance with the manufacturer’s instructions. Marc-145 cells or PAMs were grown in each well of 96-well plates to form monolayers. Difopein was added to the wells at specific concentrations, and the cells were further cultured for 48 h, following which the CCK-8 reagent was added each well. After incubation for 2 h at 37 °C, cell viability was evaluated by measuring absorbance at 450 nm. The optical density of wells containing untreated control cells was defined as indicating 100% viability. To exclude the impact of 14-3-3 overexpression on cell growth, the cell viability of Marc-145^wpxld^, Marc-145^14-3-3β^, and Marc-145^14-3-3ε^ cells were also determined by the CCK-8 assay.

### Virus titration

Marc-145 cells were seeded in 96-well plates and incubated for 24 h at 37 °C in 5% CO_2_. Virus supernatants were tenfold serially diluted and added to each well (100 μL per well) in eight repetitions. After adsorption for 1 h at 37 °C in 5% CO_2_, the medium was replaced with fresh medium containing difopein at specific concentrations. Six days after infection, the 50% cell-culture infective dose (CCID_50_) was calculated by the Reed–Muench method.

### Statistical analysis

Statistical analyses were performed by one-way analysis of variance when comparing more than two groups and Student’s *t*-test when comparing only two groups. The analyses were performed using the SPSS 20.0 software package (SPSS Inc., version 20.0; Chicago, IL, USA). The data were expressed as the mean ± standard deviation (SD) from at least three independent experiments. A *P* value < 0.05 was considered statistically significant.

## Results

### 14-3-3ε and 14-3-3β interact with NSP2

**Figure 1:**
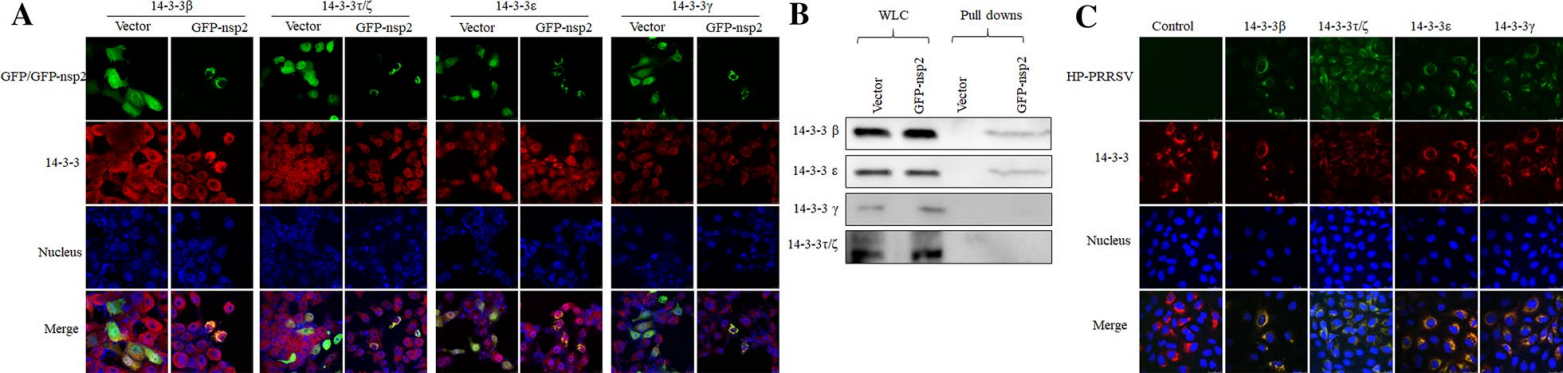
**Identification of interactions between 14-3-3 subtypes and NSP2 by confocal microscopy (A and C) and IP (B). A** Co-localization of NSP2 with 14-3-3 subtypes, confirmed by immunofluorescence microscopy. Co-localization of EGFP–NSP2 (green) with 14-3-3 β, ε, γ, and τ/ζ (red) was visualized in transfected 293T cells. **B** 293T cells were transfected with GFP–nsp2 and an empty vector. Proteins associated with NSP2 were pulled down using GFP-Trap and analyzed by Western blot using specific antibodies against 14-3-3β, ε, γ, and τ/ζ. **C** Co-localization of HP-PRRSV with 14-3-3 subtypes, confirmed by immunofluorescence microscopy. Co-localization of NSP2 of TA-12 (green) with 14-3-3 α/β, ε, γ, and τ/ζ (red) was visualized in Marc-145 cells. Co-localization was determined by the yellow signal in merged images. WCL: whole-cell lysates.

In our previous proteomics study, we had identified six subtypes of 14-3-3s as potential interactors with NSP2. To determine the specific 14-3-3 subtype that interacts with NSP2, four subtypes of 14-3-3 were selected on the basis of fold changes and peptide matches determined in the previous study (β: 5 peptides, 6.7-fold change; γ: 6 peptides, 6.17-fold change; ε: 6 peptides, 4.4-fold change; ζ: 5 peptides, 2.16-fold change; and τ: 3 peptides, 1.54-fold change) [26]. A construct containing the *nsp2* gene and an empty vector were transfected into 293T cells. At 24 h post-transfection, the cells were fixed and probed with four 14-3-3-specific antibodies or subjected to lysis for the pull-down assay and subsequent Western blot analysis. The results of both co-localization and Western blot showed that 14-3-3β and ε, but not 14-3-3γ or τ/ζ, interacted with NSP2 (Figures 1A and B). To further confirm these results, Marc-145 cells were inoculated with HP-PRRSV, and the co-localization of the virus with 14-3-3 proteins was analyzed. The results showed that all four 14-3-3 subtypes interacted with HP-PRRSV NSP2 (Figure 1C). These results suggested that 14-3-3β and ε interacted with NSP2, while 14-3-3τ/ζ/γ might interact with NSP2 indirectly by binding other cellular proteins.

### Knockdown of *14*-*3*-*3*ε decreases TA-12 infection

**Figure 2:**
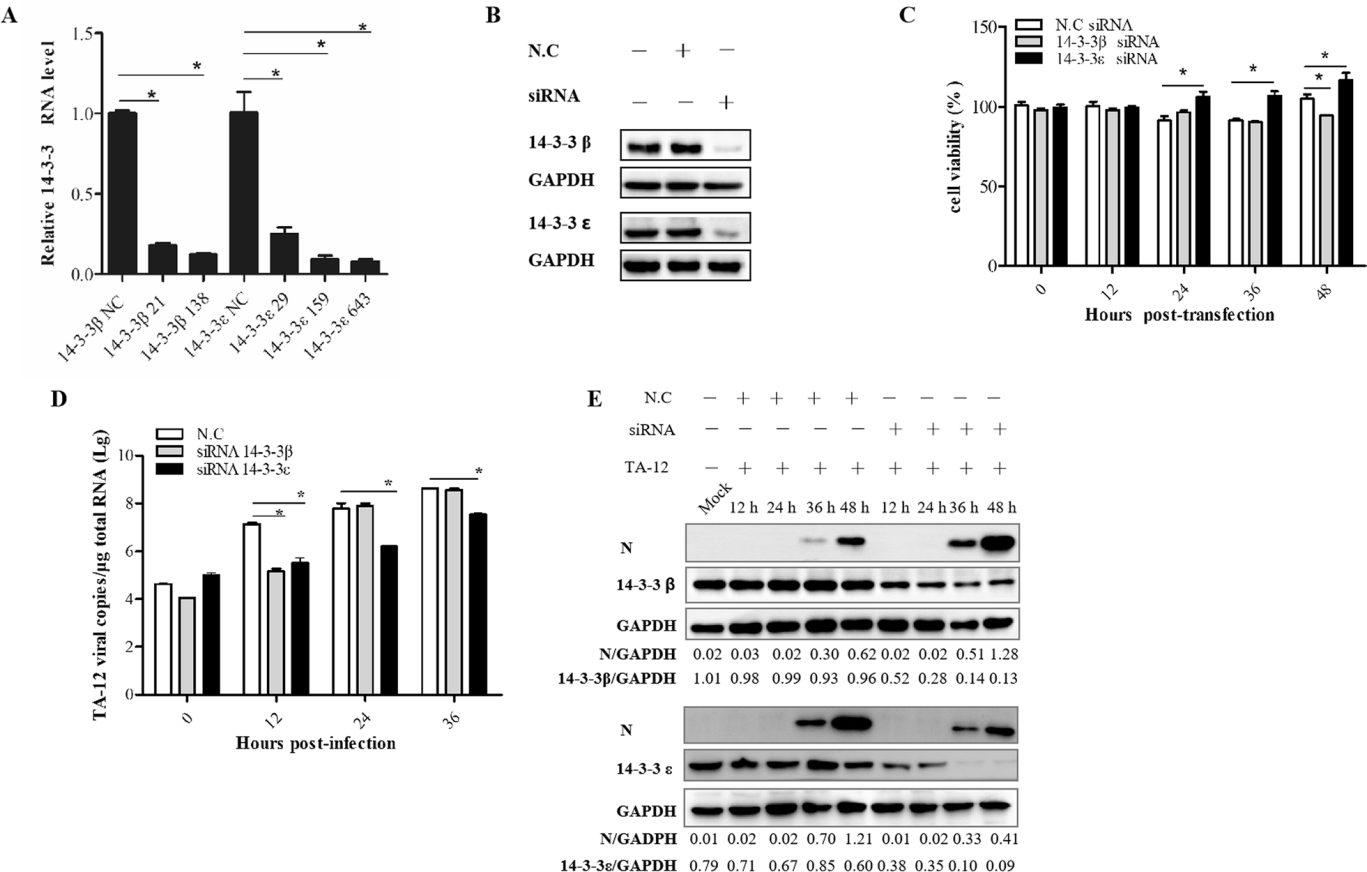
**Knockdown of** ***14*-*3*-*3ε*** **decreases HP-PRRSV infection. A** Results of qPCR for detection of *14*-*3*-*3β/ε* after siRNA transfection. Marc-145 cells were transfected with siRNA. At 24 h post-transfection, the cells were collected, and total RNA was prepared for detecting the mRNA levels of *14*-*3*-*3β/ε*. **B** Western blot analysis of 14-3-3β/ε expression after siRNA transfection. Marc-145 cells were transfected with siRNA. At 24 h post-transfection, the cells were collected, and cellular proteins were extracted for detecting 14-3-3β/ε proteins. **C** Marc-145 cells were transfected with siRNA and inoculated with TA-12 or mock infected with cell-culture medium at 24 h post-transfection. The mock-infected cells were collected at different time points after infection, and their viability was measured by the CCK-8 assay. **D** The TA-12-infected cells were collected at the same time points as the mock-infected cells and processed for total RNA extraction. Viral loads were evaluated by absolute qPCR targeting the nucleocapsid (*N*) gene of HP-PRRSV . **E** Viral proteins were evaluated by Western blot using a monoclonal antibody (6D10) targeting the PRRSV N protein. N.C: negative control. GAPDH: glyceraldehyde-3-phosphate dehydrogenase. The *GAPDH* gene and protein were used as internal controls for qPCR and Western blot. The density of the protein bands—measured with a fusion analysis software by using the VILBER lourmat imaging system (Fusion FX7, France)—was determined after subtracting the density of the GAPDH bands. *P value < 0.05.

To understand the influence of the interaction of 14-3-3β and ε with NSP2 on HP-PRRSV infection, Marc-145 cells were transfected with siRNAs targeting *14*-*3*-*3β* and ε, respectively. The results of qPCR and Western blot revealed a significant decrease in expression of both *14*-*3*-*3ε* and *14*-*3*-*3β* genes and 14-3-3ε and 14-3-3β proteins (Figures 2A and B). The CCK-8 results showed that *14*-*3*-*3ε* knockdown in Marc-145 cells increased the cell viability at 24, 36, and 48 hpi, while *14*-*3*-*3β* knockdown in Marc-145 cells decreased the cell viability only at 48 hpi (Figure 2C). This result indicated that the knockdown of the 14-3-3 subtypes had little effect on cell death (which might partly have resulted from cell apoptosis). Marc-145 cells were transfected with *14*-*3*-*3β*/*ε* siRNA and then infected with TA-12 and harvested at different time points post-infection and processed for qPCR analysis for quantifying TA-12 infection. The results showed that *14*-*3*-*3ε* knockdown caused a decrease in TA-12 replication at 12, 24, and 36 hpi, while 14-3-3β knockdown caused a decrease in viral replication only at 12 hpi (Figure 2D). Western blotting results also showed that *14*-*3*-*3ε* knockdown inhibited TA-12 infection at 36 and 48 hpi, while no such inhibition was observed in *14*-*3*-*3β*-knockdown Marc-145 cells (Figure 2E). These results indicated that knockdown of *14*-*3*-*3ε*, but not that of *14*-*3*-*3β*, caused a significant decrease in HP-PRRSV infection.

### Overexpression of 14-3-3ε enhances PRRSV replication

**Figure 3:**
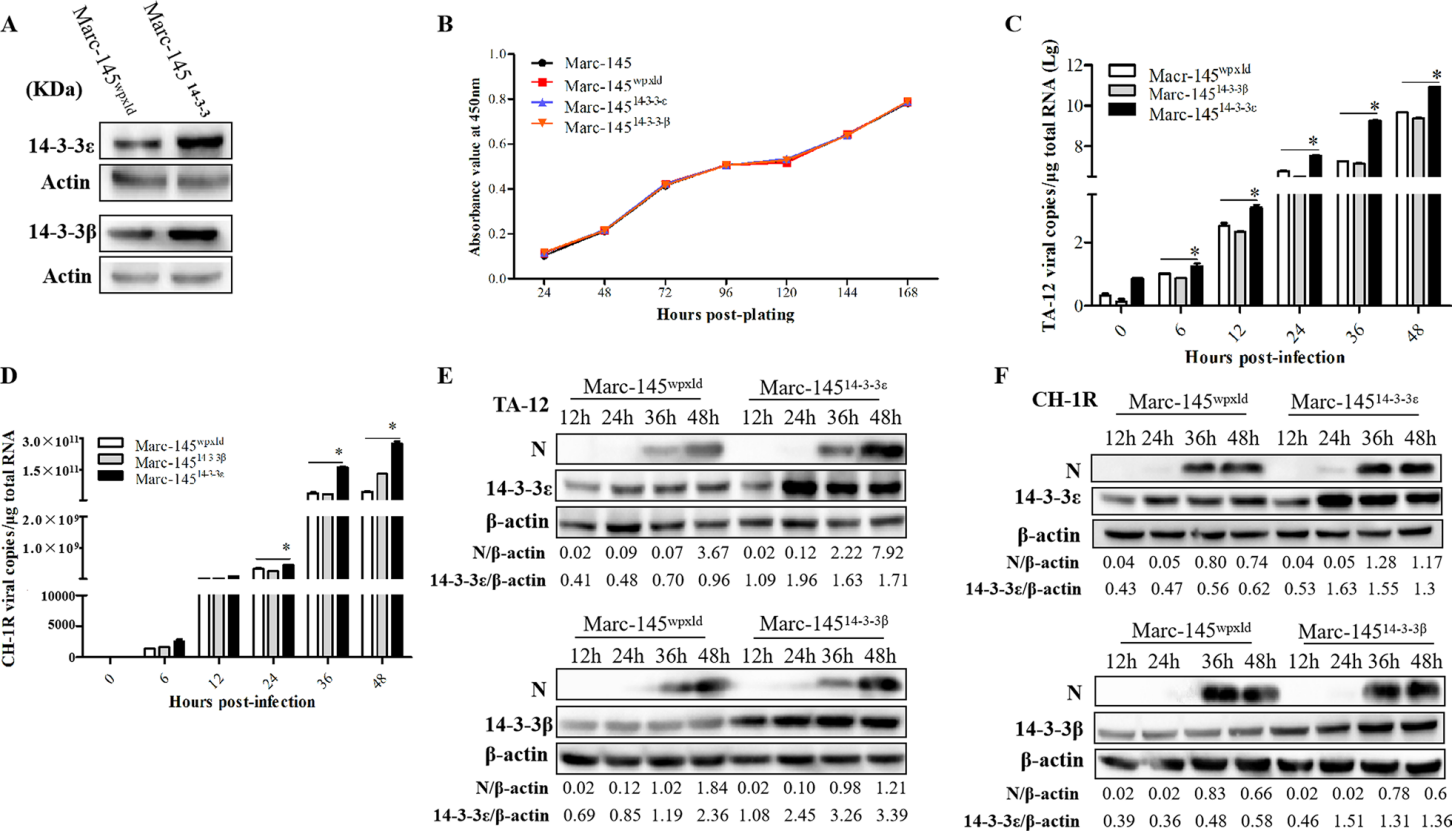
**Overexpression of 14-3-3ε enhances HP-PRRSV infection. A** Western blot analysis of 14-3-3β/ε overexpression cells. **B** Cell growth kinetics. Normal Marc-145 cells and Marc-145^wpxld^, Marc-145^14-3-3β^, and Marc-145^14-3-3ε^ cells were plated in 24-well plates. At 24, 48, 72, 96, 120, 144, and 168 h post-plating, the CCK-8 reagent was added to the cells. The cells were then incubated for 2 h at 37 °C. Cell counting was performed by measuring absorbance at 450 nm. Marc-145^14-3-3β^, Marc-145^14-3-3ε^, and Marc-145^wpxld^ cells were infected with TA-12 and CH-1R, respectively. The infected cells were harvested at 0, 6, 12, 24, 36, and 48 hpi for RNA extraction and at 12, 24, 36, and 48 hpi for protein extraction. Viral loads were evaluated by absolute qPCR targeting the *N* gene of TA-12 (**C**) and CH-1R (**D**). Cellular proteins were analyzed by Western blot analysis for detecting the viral N protein of TA-12 (**E**) and CH-1R (**F**). The GAPDH protein or *GAPDH* gene was used as the internal control. *P value < 0.05.

A lentivirus-based vector produced using a three-plasmid system was employed to develop stable 14-3-3ε/β-overexpression cell lines. Overexpression was confirmed by Western blot analysis (Figure 3A). A CCK-8 assay was performed to exclude the influence of 14-3-3 overexpression on cell growth. The results showed no significant differences in growth kinetics between normal Marc-145 cells and the 14-3-3-overexpression or empty-vector-infected cells (Figure 3B).

Upon inoculation with TA-12 and CH-1R, Marc-145^14-3-3ε^ cells exhibited higher viral copy numbers than Marc-145 cells transfected with the vector control (Marc-145^wpxld^; Figures 3C and D); in contrast, Marc-145^14-3-3β^ cells exhibited no such influence on the replication of either PRRSV strain. The results of Western blot also demonstrated a higher amount of viral protein in Marc -145^14-3-3ε^ cells but not in Marc-145^14-3-3β^ cells (Figures 3E and F). These results further confirmed that 14-3-3ε, but not 14-3-3β, can take part in PRRSV infection.

### Difopein inhibits PRRSV infection in Marc-145 cells

**Figure 4:**
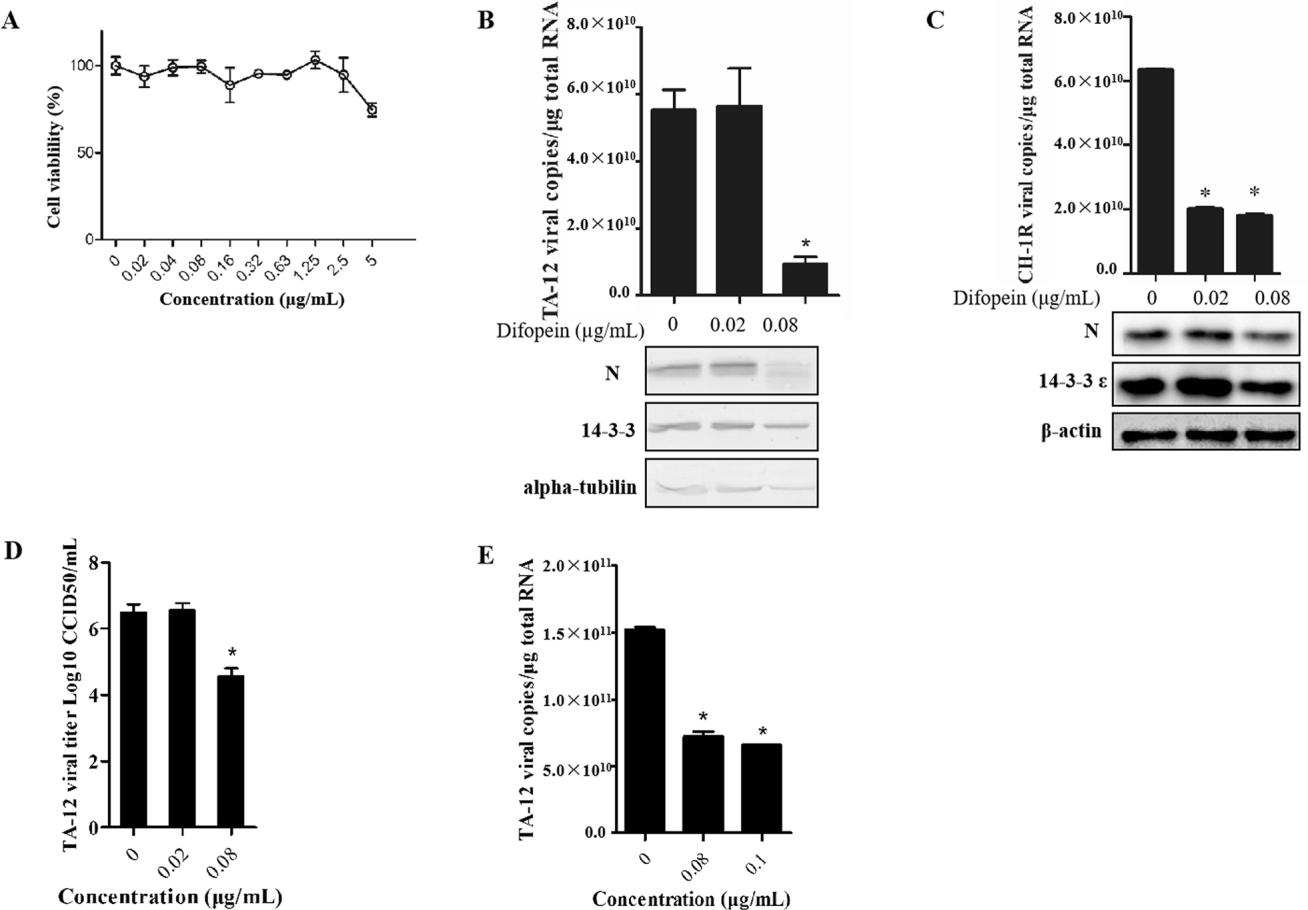
**Difopein decreases HP-PRRSV infection in Marc-145 cells. A** The cytotoxicity of difopein was determined by the CCK-8 assay. Monolayers of Marc-145 cells in 96-well plates were treated with difopein at different concentrations for 48 h, after which the CCK-8 reagent was added to each well. After incubation for 2 h, cell viability was evaluated by measuring absorbance at 450 nm. Marc-145 cells were inoculated with TA-12 and CH-1R and incubated for 1 h, after which the medium was replaced with maintenance medium containing 0.02 or 0.08 μg/mL difopein. The cells were collected 24 h later, and total RNA and cellular proteins were extracted. Viral loads were evaluated by absolute qPCR targeting the nucleocapsid (*N*) gene, and viral proteins were detected by Western blot analysis of TA-12- (**B**) and CH-1R- (**C**) infected cells. **D** CCID_50_ analysis for titration of HP-PRRSV after difopein treatment. Marc-145 cells were seeded in 96-well plates. Virus supernatants were tenfold serially diluted (range 10^2^–10^10^) and added to each well (at 100 μL per well) in eight repetitions. At 6 days post-infection, the numbers of cells in wells exhibiting cytopathic effects were counted, and CCID_50_ was calculated by the Reed–Muench method. **E** Marc-145 cells were infected with TA-12 and treated with 0.08 or 0.1 μg/mL difopein at 24 hpi. After incubation for another 12 h, the cells were collected for RNA extraction and qPCR analysis. *P value < 0.05.

Difopein is a dimeric version of the R18 peptide, which binds to 14-3-3 proteins with high affinity. It competitively inhibits 14-3-3–ligand interactions and hinders the ability of 14-3-3 to bind target proteins. The results of the CCK-8 assay demonstrated the low cytotoxicity of difopein in Marc-145 cells (Figure 4A). Shortly after infection with TA-12 and CH-1R, Marc-145 cells were treated with difopein at concentrations of 0, 0.02, and 0.08 μg/mL. The cells were harvested at 24 hpi and analyzed by qPCR and Western blot. The results showed that 0.08 μg/mL difopein caused a significant decrease in infection by both strains (Figures 4B and C). In CH-1R-infected cells, the copy number of the viral genome had decreased significantly, as indicated by the qPCR results (Figure 4C). The extent of TA-12 infection was evaluated by a virus-titration assay, which revealed that 0.08 μg/mL difopein caused a 1-log decrease in TA-12 infection relative to the viral titer observed in non-treated cells (Figure 4D). To confirm the therapeutic property of difopein, the drug was added to Marc-145 cells 24 h after inoculation with TA-12 or CH-1R. The qPCR results showed that treatment with 0.08 and 0.1 μg/mL difopein caused a decrease in the TA-12 copy number (Figure 4E). These results indicated that difopein treatment caused a significant prophylactic decrease in TA-12 replication and thus exerted a therapeutic effect against TA-12 infection.

### *14*-*3*-*3*ε knockdown and difopein treatment decrease HP-PRRSV infection in PAMs

**Figure 5:**
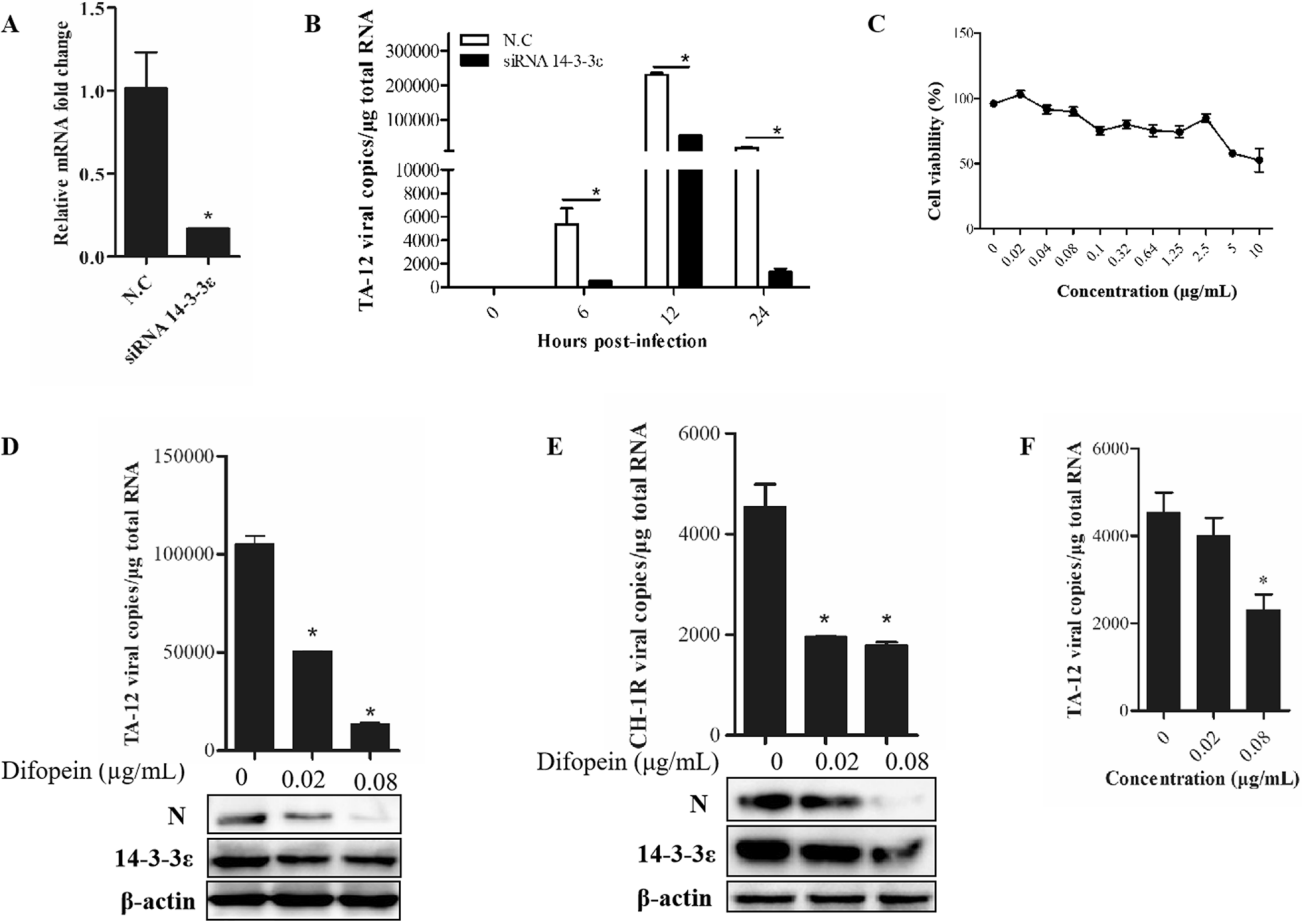
***14*-*3*-*3ε*** **knockdown and difopein treatment decrease HP-PRRSV infection in PAMs. A** Results of qPCR for detection of *14*-*3*-*3ε* after knockdown. PAMs were transfected with siRNA. The cells were collected at 24 h post-transfection, and total RNA was prepared for detecting the mRNA levels of *14*-*3*-*3ε*. **B** Results of qPCR for analysis of TA-12 replication after *14*-*3*-*3ε* knockdown. PAMs were transfected with siRNA and inoculated with TA-12 at 24 h post-transfection. The cells were collected, and total RNA was extracted. Viral loads were evaluated by absolute qPCR targeting the *N* gene of TA-12. **C** The cytotoxicity of difopein in PAMs was determined by the CCK-8 assay. PAMs were inoculated with TA-12 or CH-1R and incubated for 1 h, after which the medium was replaced with maintenance medium containing 0.02 or 0.08 μg/mL difopein. The cells were collected 24 h later, and total RNA was extracted. Viral loads were evaluated by absolute qPCR targeting the *N* gene, and viral proteins were detected by Western blot analysis of TA-12- (**D**) CH-1R- (**E**) infected cells. **F** Therapeutic role of difopein in TA-12 infection. PAMs were infected with TA-12 and treated with 0.02 or 0.08 μg/mL difopein at 24 hpi. The cells were incubated for another 12 h and then harvested for RNA extraction and qPCR analysis.

Alveolar macrophages are the main target cells of PRRSV in vivo. In this study, PAMs were transfected with *14*-*3*-*3*ε siRNA in order to determine the effect of *14*-*3*-*3*ε knockdown on HP-PRRSV infection in PAMs. The qPCR results revealed an approximately 85% knockdown of *14*-*3*-*3ε* mRNA expression (Figure 5A). The *14*-*3*-*3ε* knockdown had significant effects on PRRSV infection. Relative to the control, the copy numbers of PRRSV in *14*-*3*-*3ε*-knockdown PAMs had decreased by 90.9%, 76.5%, and 92.9% at 6, 12, and 24 hpi, respectively (Figure 5B).

The cell viability of PAMs after difopein treatment was evaluated by the CCK-8 assay (Figure 5C). On the basis of the cell viability findings, difopein was added to PAMs at concentrations of 0.02 and 0.08 μg/mL before TA-12 or CH-1R infection or after infection. The results showed that treatment of PAMs with 0.02 and 0.08 μg/mL difopein before TA-12 replication caused a decrease of 52% and 87% in TA-12 copy numbers, respectively. The decrease in viral N protein expression was confirmed by Western blot (Figure 5D). The copy numbers of CH-1R had also decreased significantly after difopein treatment at concentrations of 0.02 and 0.08 μg/mL, as indicated by the results of qPCR and Western blot (Figure 5E). The therapeutic effects of difopein were analyzed in PAMs 24 h after TA-12 infection. The qPCR results showed that difopein caused a decrease in TA-12 infection at a concentration of 0.08 μg/mL but had no effect at a concentration of 0.02 μg/mL (Figure 5F).

## Discussion

The 14-3-3 proteins are involved in many physiological and pathological cellular processes by virtue of their interactions with a multitude of targets. They interact with client proteins and influence their activity, localization, stability, or protein–protein interactions (PPIs) and, consequently, have an effect on virus infection [20, 25, 29]. The 14-3-3 proteins are generating significant interest as potential drug targets by allowing the targeting of specific pathways to elicit therapeutic effects. Leucine-rich repeat kinase 2, one of the PPI partners of 14-3-3, could serve as a target for the development of neuroprotective therapies [30]. siRNA-mediated knockdown of *14*-*3*-*3β* arrests tumorigenesis and astrocytoma progression [31]. Knockdown of *14*-*3*-*3ζ* inhibits cancer-cell growth and, therefore, offers a therapeutic target for cancer [32, 33]. These results highlight the potential of 14-3-3s as pharmaceutical targets.

The 14-3-3 proteins have been reported to play a role in viral infection by evading the innate immune system or other cellular signaling processes [23, 34]. The 14-3-3s are also important biomarkers for nervous-system diseases caused by infection with HIV or influenza virus [19, 20, 35, 36]. However, little information is available on the possibility of 14-3-3 as a potential therapeutic candidate against viral infection. On the basis of previous findings on the interaction of PRRSV NSP2 with 14-3-3, we confirmed that 14-3-3 could be a therapeutic candidate against HP-PRRSV infection [26].

There are seven known mammalian isoforms of 14-3-3, all of which possess relatively conserved sequences (60–87% amino-acid homology) and a well-conserved structure [37]. We have previously determined 14-3-3 s to be potential interactors with the NSP2 protein of LP-PRRSV (CH-1R strain), HP-PRRSV (SD16 strain), and a type 1 PRRSV engineered by reverse genetics (SD01-08; PRRSV-NSP2-GFP). These results indicated that the 14-3-3–NSP2 interaction might be a common occurrence among different types of PRRSVs. However, this interaction might be isoform-dependent because of the functional specificity of the 14-3-3 isoforms. Therefore, the present study attempted to first identify the specific subtypes that interact with NSP2. The results of co-localization and pull-down assays both showed that 14-3-3β and ε interacted with NSP2 (Figure 1). Therefore, we did not conduct any further research on other 2 subtypes. The 14-3-3 proteins usually exist as homo- or hetero-dimers by virtue of interactions between the N-terminal regions of identical or different isoforms [38, 39]. On the basis of this information, we deduced that 14-3-3β and ε heterodimers interacted with NSP2.

Each subtype of 14-3-3 has a special function. To understand the interaction of 14-3-3β/ε with NSP2 in greater depth, we knocked down the corresponding genes using siRNA techniques. Because 14-3-3 s can regulate the cell cycle and inhibit apoptosis [16, 40, 41], we evaluated the effects of 14-3-3 knockdown on Marc-145 cells. In *14*-*3*-*3ε*-knockdown cells infected with TA-12, the cell viability had increased at 24 and 36 hpi, while the virus replication had decreased. In *14*-*3*-*3β*-knockdown cells, the cell viability had decreased at 48 hpi, while the virus replication had remained unaffected (Figures 2C–E). On the basis of these results, we concluded that the decrease in viral replication was not caused by cell apoptosis.

To further confirm the role of 14-3-3 s in PRRSV infection, *14*-*3*-*3β/*ε genes were knocked down in primary PAMs; these results also indicated that only 14-3-3ε (and not 14-3-3β) played a role in TA-12 and CH-1R replication. In contrast, overexpression of 14-3-3ε, and not 14-3-3β, in Marc-145 cells caused a significant increase in TA-12 and CH-1R replication. These results together indicated that 14-3-3ε could serve as a therapeutic candidate against PRRSV infection. The probable mechanism might be related to the important role that 14-3-3ε plays in innate immunity during infection by hepatitis C virus and other pathogenic RNA viruses by facilitating a stable RIG-I translocon [23]. However, the actual mechanism of action of 14-3-3ε in mediating HP-PRRSV infection needs further investigation.

Difopein is an inhibitor of 14-3-3 function; it exerts its inhibitory activity by competitively blocking 14-3-3 PPIs. In this study, difopein was evaluated for its antiviral activity by addition to PRRSV-infected Marc-145 cells or PAMs. The effects of difopein on PRRSV infection were evaluated before and after PRRSV replication. All corresponding results showed that difopein caused a significant decrease in TA-12 infection (Figures 4B, D, E and 5B, D, F). Because of the universal inhibitory capability of difopein—arising from competition with 14-3-3 s for binding target proteins—the decrease in PRRSV infection after difopein treatment might be a result of the drug blocking the interaction of 14-3-3τ/ζ/γ with other viral proteins, which were found to be co-localized with NSP2 (Figure 1C). We, therefore, deduced that difopein has a universal inhibitory effect on PRRSV infection; however, this hypothesis needs to be confirmed by further research. However, the present data showed that inhibition of 14-3-3ε function contributed greatly to decreasing TA-12 replication through interaction with NSP2.

In conclusion, the present study demonstrated that inhibiting the interaction of 14-3-3ε with its ligands decreased TA-12 and CH-1R replication. This suggests that 14-3-3ε could be used as a therapeutic candidate against PRRSV infection. Decreased expression or inhibition of 14-3-3ε inhibited PRRSV replication. Thus, siRNA-mediated *14*-*3*-*3ε* knockdown and difopein treatment might be useful prevention and treatment strategies against PRRSV infection and warrant further in vivo evaluation.
